# RF energy harvesting schemes for intelligent reflecting surface-aided cognitive radio sensor networks

**DOI:** 10.1038/s41598-022-26853-0

**Published:** 2022-12-28

**Authors:** Jihong Wang, Hongquan Yu

**Affiliations:** School of Electrical Engineering, Northeast Electric Power University, Jilin, 132012 China

**Keywords:** Energy harvesting, Information technology, Electrical and electronic engineering

## Abstract

Energy harvesting (EH) is a potential solution to enhance the node sustainability and prolong the network lifetime of cognitive radio sensor networks (CRSNs). However, CRSNs nodes can only harvest energy from the direct link with energy sources, and severe path loss results in low energy utilization ratio. To solve the above problem, intelligent reflecting surface (IRS) is introduced, and a shared reflection coefficient matrix-based EH scheme is proposed for IRS-aided CRSNs in this paper. An optimization problem with the objective of maximizing the total amount of energy harvested by all CRSNs nodes is formulated, and by optimally adjusting the IRS reflection coefficient, CRSNs nodes can harvest energy from both the direct link and the cascaded reflection link via IRS, which increases the amount of harvested energy. In addition, a subsurface partition-based EH scheme is proposed to reduce the additional computational complexity brought by increasing IRS elements or CRSNs nodes. Simulation results show that the proposed schemes can both dramatically improve energy utilization ratio, and the subsurface partition-based EH scheme will bring in less than 1 percent performance loss when compared with the other scheme, i.e., reasonable subsurface partition can achieve a balance between harvested energy and computational complexity.

## Introduction

Cognitive radio sensor networks (CRSNs) are smart combination of legacy wireless sensor networks (WSNs) and cognitive radio (CR) technology. Idle licensed spectrum can be leveraged for communication in an opportunistic manner through dynamic spectrum sensing and spectrum access^1^, and the spectrum shortage faced by legacy WSNs can be effectively mitigated^2,3^. However, CRSNs nodes are usually powered by limited-capacity battery which cannot be replaced periodically. Performing CR functions such as periodical spectrum sensing and decision is pretty energy-consuming, which will result in fast energy exhaustion, and the network lifetime will be shortened^4^. Energy harvesting (EH) technique enables nodes to harvest energy from natural sources such as solar and wind or radio frequency (RF) sources to compensate for limited battery energy and prolong their lifetime^5,6^. Therefore, EH is a promising solution to solve the energy constraint problem of CRSNs^7,8^, and it has the potential of guaranteeing stable and sustainable network operation.

Compared with instable and expensive natural sources, RF energy sources can provide ubiquitous, relatively stable and predictable energy supplement for CRSNs nodes, as a result, RF EH attracts widespread attention and has been widely applied in practice^9,10^. In RF EH-CRSNs, the sink acts as a dedicated energy source to radiate RF signal, and CRSNs nodes harvest energy from the RF signal received from the downlink. The harvested energy is a supplement of battery energy and is stored for future use. In addition, according to the Euclidean distance to the sink and the minimum received signal to noise ratio (SNR) required to guarantee successful information decoding at the sink, CRSNs nodes adjust their transmission power to deliver their sensed data towards the sink in the uplink. However, as CRSNs nodes harvest energy and transmit data only through the direct link with the sink, severe path loss will limit the energy harvested by nodes far away from the sink^11^ and require high transmission power to reach the sink. Limited harvested energy and high energy consumption will accelerate node energy exhaustion and shorten the network lifetime. In order to solve this problem, intelligent reflecting surface (IRS) technique is introduced to construct an extra cascaded reflection link. Each IRS element can adjust the amplitude and phase shift of the incident signal independently, and in this case, signal received from the direct link and the cascaded reflection link via IRS can be coherently combined to improve the received power^12^. To be specific, assisted by IRS, CRSNs nodes can harvest RF energy from both the direct link and the cascaded reflection link, which can increase the amount of harvested energy and improve the energy utilization ratio. On the other hand, assisted by the extra transmission link via IRS, CRSNs nodes can lower their transmission power while satisfying the minimum received SNR at the sink, which will reduce node energy consumption and expand node lifespan. In a word, IRS-aided EH-CRSNs have great potentials to better exploit EH to compensate for limited node battery and conserve energy, which is beneficial for significantly extending the network lifetime.

To further explore the impact of IRS deployment and reflection coefficient matrix configuration on EH performance, EH schemes which can maximize the total amount of energy harvested by all CRSNs nodes are proposed for IRS-aided EH-CRSNs in this paper, and the innovations are summarized as follows:

- IRS is introduced into RF EH-CRSNs to help enhance the energy utilization ratio during EH, and the optimization problem with the objective of maximizing the total amount of energy harvested by all CRSNs nodes is formulated. Successive convex approximation (SCA) algorithm is applied to solve it and implement the shared reflection coefficient matrix-based EH scheme.
- To reduce the additional computational complexity caused by increasing IRS elements or CRSNs nodes and achieve an effective compromise between harvested energy and computational complexity, a subsurface partition-based EH scheme is proposed, and the relationship between network performance and the number of subsurface is explored. Simulation results show that the above EH schemes can both significantly increase the amount of harvested energy. In addition, the subsurface partition-based EH scheme further reduces the computational complexity at the cost of less than 1% performance loss.

## Related works

As research on designing EH schemes for IRS-aided EH-CRSNs is still in its infancy, related research results in IRS-aided EH-nonCRSNs and EH-CRSNs without IRS are reviewed in this section to exhibit the differences between our works and the existing ones.

### Research on IRS-aided EH-nonCRSNs

Recently, IRS is widely applied to assist in enhancing the EH performance of various wireless communication systems, such as IRS-aided simultaneous wireless information and power transfer (SWIPT) system, wireless powered communication networks (WPCNs) and other systems.

In IRS-aided SWIPT system, the base station (BS)/access point (AP) transfers data and energy simultaneously through the downlink. To be specific, by jointly optimizing the transmitting pre-coding matrix of multi-antenna AP and IRS reflection coefficient matrix, while satisfying the constraint of harvested energy at energy users, objectives such as minimizing the transmission power of AP^13^, maximizing the weighted sum of power received by all energy users^14^ and maximizing the weighted sum rate of all information users^15^ are achieved, respectively. In order to better exhibit the actual characteristics of IRS-aided SWIPT system, the impact of incidence angle and reflection angle of electromagnetic wave on IRS reflection is further explored based on non-linear EH model^16^. IRS grouping is utilized to achieve a compromise between the system performance and computational complexity. In addition, IRS reflection coefficient matrix and BS beamforming vector are jointly optimized to minimize the transmission power of BS. Non-linear EH model and power splitting ratios of all users are taken into consideration, and the energy beamforming at BS, IRS reflection coefficient matrix and power splitting ratios of all users are jointly optimized^17^. The maximum-minimum system energy efficiency is maximized by leveraging penalty-based algorithm and inner approximation-based algorithm to guarantee the energy efficiency fairness among users. Concentrating on the security performance of IRS-aided SWIPT system, artificial noise is introduced at AP, and the noise covariance matrix, AP beamforming vector and IRS reflection coefficient matrix are jointly optimized to maximize the system energy efficiency^18^. By applying power splitting at the receiver, the same optimization manner is utilized to maximize the achievable user security rate, suppress inter-user interference and balance the harvested energy among users^19^. In IRS-aided EH-CRSNs, the main traffic type is uplink data transmission from CRSNs nodes towards the sink, which is quite different from IRS-aided SWIPT system. Different traffic types determine that the above algorithms cannot be applied to IRS-aided EH-CRSNs.

In IRS-aided WPCNs, users harvest energy from the downlink and completely rely on the harvested energy to carry out uplink data transmission. Concentrating on IRS-aided WPCNs, alternating optimization algorithm is leveraged to optimize the IRS reflection coefficient matrix^20^. In this case, the EH efficiency of each device is improved, and the harvested energy is further used to transmit data towards the data BS. A non-linear EH model-based optimization problem is formulated for WPCNs, and the total transmission energy of power station (PS) is minimized by jointly optimizing the active and passive beamforming^21^. Based on the same non-linear EH model, power-splitting receiver architecture with multiple rectifiers is designed to avoid the received power from falling into saturation^22^. The energy conversion efficiency of EH circuits is promoted through interference suppression at the receiver. A safe and intelligent EH framework is proposed for 6G Internet of Things, and with the assistance of IRS, energy users can leverage the energy harvested from ambient environment to transmit the processed data towards the BS^23^. In addition, IRS-aided EH satisfaction is modeled according to users’ energy requirement, and a deep reinforcement learning-based resource allocation scheme is proposed with the purpose of maximizing the weighted sum of user satisfaction to enhance the EH efficiency. In IRS-aided EH-CRSNs, the energy harvested from the downlink is regarded as a supplement of the battery energy, and it will not impose energy causality constraint on uplink data transmission, which is totally different from IRS-aided WPCNs. Different objectives and constraints determine that the research results of IRS-aided WPCNs cannot be applied to IRS-aided EH-CRSNs.

Apart from the above research results, IRS is also applied to wireless power transfer (WPT) system and unmanned aerial vehicle communication system. Active and passive beamforming are jointly designed for IRS-aided WPT system to extend EH range of the whole network^24^. Considering the scenario where there exist eavesdroppers around legitimate users, the optimization problem with the objective of maximizing the system security rate and minimizing the energy harvested by eavesdroppers is formulated^25^. To conquer the limitations of fixed energy sources and increase the flexibility of energy supply, drones act as energy sources to emit RF signal^26^. The trajectory and transmission power of drones and IRS reflection coefficient matrix are jointly designed to maximize the sum rate of the whole system. In IRS-aided EH-CRSNs, the sink which acts as both energy source and data aggregation point is fixed, which is different from IRS-aided unmanned aerial vehicle communication system where position of drones keeps changing. Different characteristics and aims determine that the above research results cannot be applied to IRS-aided EH-CRSNs either. In order to show their differences more clearly, the characteristics of the above works are summarized in Table 1 below, and “√” and “ × ” represent whether they possess corresponding characteristics.

**Table 1:** Characteristics analysis of related works.

References	Application scenarios	Objective functions	Whether IRS is partitioned into subsurface	Whether the harvested energy is used to supplement node battery energy
^13^	IRS-aided SWIPT system	Minimize the transmission power of AP	×	×
^14^	IRS-aided SWIPT system	Maximize the weighted sum of received power at all energy users	×	×
^15^	IRS-aided SWIPT system	Maximize the weighted sum rate of all information users	×	×
^16^	IRS-aided SWIPT system	Minimize the transmission power of BS	√	×
^17^	IRS-aided SWIPT system	Maximize the maximum-minimum system energy efficiency	×	×
^18^	IRS-aided SWIPT system	Maximize the system energy efficiency	×	×
^19^	IRS-aided SWIPT system	Maximize the achievable user security rate	×	×
^20^	IRS-aided WPCNs	Maximize the weighted sum rate of all information users	×	×
^21^	IRS-aided WPCNs	Minimize the total transmission energy of PS	×	×
^22^	IRS-aided WPCNs	Minimize the energy consumption of hybrid AP	×	×
^23^	6G Internet of Things	Maximize the weighted sum of user satisfaction	×	×
^24^	IRS-aided WPT system	Maximize EH range of the whole system	×	×
^25^	IRS-aided wireless system	Maximize the system security rate and minimize the energy harvested by eavesdroppers	×	×
^26^	IRS-aided unmanned aerial vehicle communication system	Maximize the sum rate of the whole system	×	√

### Research on EH-CRSNs without IRS

Existing research on EH-CRSNs mainly focuses on resource allocation and management. A medium access control protocol is proposed to reduce collisions among nodes and improve the system throughput^27^. EH is leveraged to compensate for battery energy and enhance node sustainability to prolong the network lifetime. Differential evolution algorithm is utilized to optimize user satisfaction of the whole EH-CRSNs and improve energy efficiency and spectral efficiency^28^. An optimal node paring and channel matching strategy is proposed for RF EH-CRSNs^29^, and according to node residual energy and channel availability, K-means clustering-based two-level classification algorithm is leveraged to divide nodes into categories to perform different tasks, which is beneficial for balancing the residual energy among nodes and facilitating successful data delivery. Aiming at conquering the limitations of solely concentrating on intra-cluster communication, an effective energy and channel assignment strategy is proposed to solve intra-cluster and inter-cluster energy and channel management problem^30^. However, each node is configured with 2 antennas for EH and data delivery, respectively, which is not applicable to single-antenna CRSNs nodes. A sub-channel and resource allocation strategy based on spectrum lease is proposed for CRSNs, and outage probability is taken into consideration to ensure network robustness in complex environment^31^. However, it is only suitable for single-hop CRSNs. A multi-hop clustering routing protocol based on non-linear RF EH is proposed, and energy control mechanism is introduced to manage node status^32^. In addition, energy level function-based cluster heads and relay selection criteria are defined to enhance the node sustainability and extend the network lifetime. However, nodes in the above clustering protocols and channel allocation strategies can only harvest energy from the direct link with energy sources. Signal propagation will suffer severe path loss, and the total amount of harvested energy is heavily constrained. In addition, without considering IRS reflection coefficient matrix configuration, the above research results cannot be applied to IRS-aided EH-CRSNs.

## RF EH schemes designed for IRS-aided EH-CRSNs

As each IRS element is intelligently programmable, IRS reflection coefficient matrix configuration will affect the amount of energy harvested by CRSNs nodes. In this paper, 3 RF EH schemes are proposed for IRS-aided EH-CRSNs to reasonably configure the reflection coefficient matrix, i.e., time division-based EH scheme, shared reflection coefficient matrix-based EH scheme and subsurface partition-based EH scheme. In the following subsections, system model is firstly presented and then the 3 proposed RF EH schemes are elaborated.

### System model

**Figure 1:**
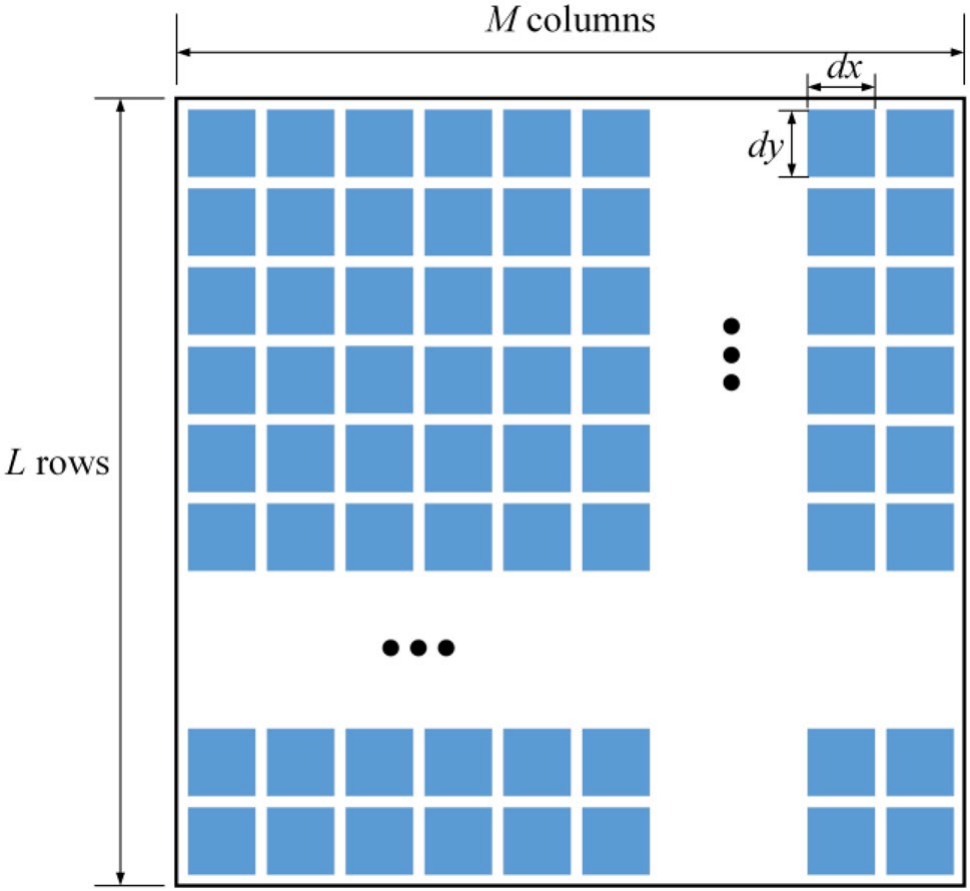
The schematic diagram of IRS architecture.

*K* homogeneous CRSNs nodes (denoted by set ***K*** = {1,2,…,*K*}) are randomly distributed in the square monitoring area with size *A* × *A*. The single-antenna sink is located at the center and it serves as a dedicated energy source to emit RF signal with fixed transmission power *P*_0_. With the assistance of IRS, CRSNs nodes can harvest energy from the direct link and the cascaded reflection link via IRS simultaneously to compensate for their limited battery energy, i.e., RF signal received from the sink is converted by internal circuits with conversion efficiency *α* and stored into battery for future use. As shown in Fig. 1, IRS is composed of *N* elements (denoted by set ***N***) which are regularly arranged into *L* rows and *M* columns, i.e., *N* = *L* × *M*, and the size of each IRS element is *dx* × *dy*. Intervals between neighboring IRS elements are ignored. The IRS reflection coefficient matrix is expressed as **θ** = diag(*θ*_1_,…,*θ*_*n*_,…,*θ*_*N*_), here, $\theta_{n} = \beta_{n} e^{{j}{\xi_{n} }}$, and *β*_*n*_ and *ξ*_*n*_ represent the reflection amplitude and phase shift of the *n*th IRS element, respectively. To maximize the total amount of harvested energy, the optimal reflection amplitude of the *n*th IRS element is set as *β*_*n*_ = 1.

**Figure 2:**
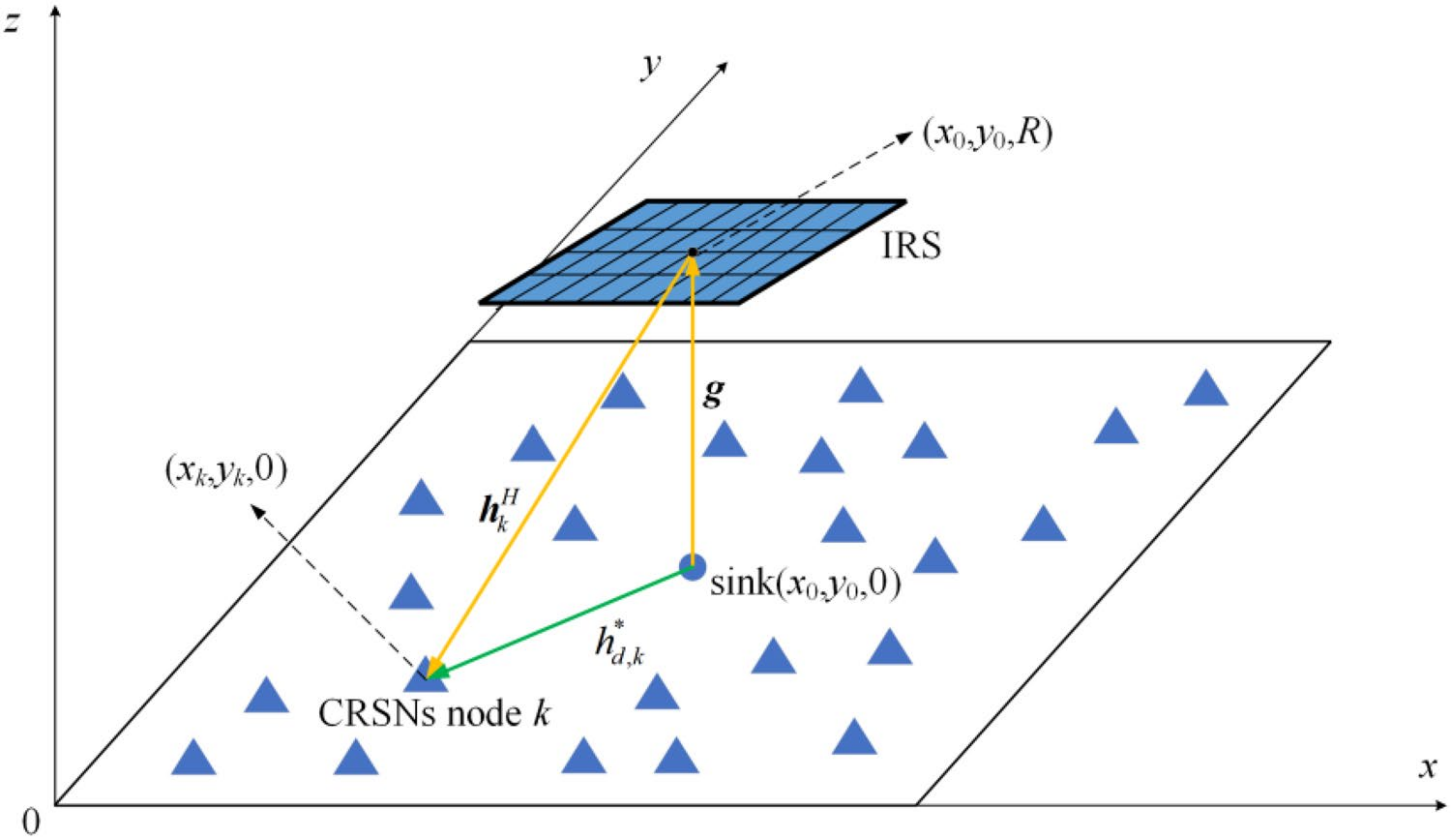
Network architecture of IRS-aided EH-CRSNs.

It has been demonstrated that IRS should be deployed close to the transmitter or the receiver^33^. To ensure that all CRSNs nodes can enjoy the array gain brought by IRS, IRS is deployed just above the sink (i.e., the transmitter) in this paper. Cartesian coordinate system is established as shown in Fig. 2, and all CRSNs nodes and the sink are located on the *x*–*y* plane. The coordinate of the sink is (*x*_0_,*y*_0_,0), while CRSNs node *k*(∀*k* ∈ ***K***) is located at (*x*_*k*_,*y*_*k*_,0). IRS is deployed parallel to the *x*–*y* plane with vertical distance *R*, and the coordinate of its center is denoted by (*x*_0_,*y*_0_,*R*). To establish a performance upper bound for the considered system, the instantaneous channel status information (CSI) of the entire system is assumed to be available at the sink. Acquiring instantaneous CSI is not in the scope of this paper, and it can be acquired with one of the existing channel estimation schemes proposed for IRS-aided wireless systems. The equivalent channels between the sink and IRS, between IRS and CRSNs node *k*, between the sink and CRSNs node *k* are represented by ${\varvec{g}} \in {\mathbb{C}}^{N \times 1}$, ${\varvec{h}}_{k}^{\rm {H}} \in {\mathbb{C}}^{1 \times N}$ and $h_{d,k}^{*}$, respectively.

### Time division-based EH scheme for IRS-aided EH-CRSNs

The time division-based EH scheme divides the total EH time duration *T* evenly into *K* time slots and assigns a dedicated time slot to each CRSNs node to enable the optimal EH. Each CRSNs node harvests energy from the RF signal emitted by the sink whose transmission power is *P*_0_, and its received signal power, i.e., the input power of the EH circuits of node *k P*_*in*_(*k*) is:

1 $$P_{in} (k) = P_{0} \times \left| {{\varvec{h}}_{k}^{\rm {H}} {{{\varvec{\uptheta}}}}_{k} {\varvec{g}}{ + }h_{d,k}^{*} } \right|^{2}$$

In this paper, linear EH is adopted to quantify the energy harvested by CRSNs nodes, that is, the output power of the EH circuits will increase linearly with the input power, and the conversion efficiency is *α*. Therefore, the harvested power of CRSNs node *k P*_*EH*_(*k*) is:

2 $$P_{EH} (k) = \alpha \times P_{0} \times \left| {{\varvec{h}}_{k}^{\rm {H}} {{\varvec{\uptheta}}}_{k} {\varvec{g}}{ + }h_{d,k}^{*} } \right|^{2}$$

Each CRSNs node harvests energy from the sink within its dedicated time duration *T/K*, and the amount of energy harvested by node *k E*_*EH*_(*k*) is:

3 $$E_{EH} (k) = \frac{T}{K} \times \alpha \times P_{0} \times \left| {{\varvec{h}}_{k}^{\rm {H}} {{\varvec{\uptheta}}}_{k} {\varvec{g}}{ + }h_{d,k}^{*} } \right|^{2}$$

The total amount of RF energy harvested by all CRSNs nodes *E*_*total*_ is the summation of the amount of energy harvested by each node, as shown below:

4 $$E_{total} = \sum\limits_{k = 1}^{K} {E_{EH} (k)} = \sum\limits_{k = 1}^{K} {\frac{T}{K} \times \alpha \times P_{0} \times } \left| {{\varvec{h}}_{k}^{\rm {H}} {{\varvec{\uptheta}}}_{k} {\varvec{g}}{ + }h_{d,k}^{*} } \right|^{2}$$

The optimization problem with the objective of maximizing the total amount of harvested energy can be formulated as:

5 $$\rm {(P}1):\quad \begin{array}{*{20}c} {\mathop {\max }\limits_{{\left\{ {{{\varvec{\uptheta}}}_{k} } \right\}}} } & {\sum\limits_{k = 1}^{K} {\frac{T}{K} \times \alpha \times P_{0} \times } \left| {{\varvec{h}}_{k}^{\rm {H}} {{\varvec{\uptheta}}}_{k} {\varvec{g}}{ + }h_{d,k}^{*} } \right|^{2} } \\ \end{array}$$

6 $${\text{s.t.}} \quad 0 \le \xi_{n,k} \le 2\pi ,\forall n \in {\varvec{N}}$$

The inherent properties of the optimization problem can be exploited to obtain high-quality solutions for it. In the time division-based EH scheme, IRS can be reconfigured *K* times to serve each individual CRSNs node *k* in its dedicated time slot. In other words, the IRS reflecting coefficient matrix for node *k* has nothing to do with that for others. In this case, the optimization problem can be decomposed into *K* sub-problems which can be solved independently. According to^33^, the optimal IRS reflection coefficient matrix for node *k* should guarantee the coherent combination of the RF signal received from the direct link and the cascaded reflection link via IRS to maximize its total amount of harvested energy. Correspondingly, the optimal reflecting coefficient of the *n*th IRS element for node *k* is:

7 $$\xi_{n,k}^{*} = \bmod (\varsigma_{k} - (\phi_{n,k} + \zeta_{n} ),2\pi )$$

where *ϕ*_*n*,*k*_, *ζ*_*n*_ and *ς*_*k*_ are the phase shift introduced by the signal propagation between the *n*th IRS element and node *k*, between the sink and the *n*th IRS element and between the sink and node *k*, respectively.

The time division-based EH scheme enables each CRSNs node to possess its dedicated EH time slot, and IRS is fully leveraged to serve it during this time. However, the EH duration assigned to each node is so short that the harvested energy is restricted. In addition, path loss will deteriorate as the propagation distance increases. Therefore, the energy harvested by CRSNs nodes far away from the sink is rather limited.

### Shared reflection coefficient matrix-based EH scheme for IRS-aided EH-CRSNs

To conquer the limitations of the time division-based EH scheme, the shared reflection coefficient matrix-based EH scheme enables all CRSNs nodes to harvest energy from both the direct link and the cascaded reflection link via IRS during *T*. An optimization problem with the objective of maximizing the total amount of energy harvested by all CRSNs nodes is also formulated, and the optimal IRS reflection coefficient matrix can be derived by solving it.

In the shared reflection coefficient matrix-based EH scheme, the total amount of energy harvested by all CRSNs nodes is:

8 $$E_{total}^{\prime } = \sum\limits_{k = 1}^{K} {T \times \alpha \times P_{0} \times \left| {{\varvec{h}}_{k}^{\rm {H}} {{\varvec{\uptheta}}}{\varvec{g}}{ + }h_{d,k}^{*} } \right|^{2} }$$

The optimization problem with the objective of maximizing the total amount of harvested energy can be formulated as:

9 $$\hbox {(P}2):\quad \mathop {\max }\limits_{{{\varvec{\uptheta}}}} \quad \sum\limits_{{\it{k}} = 1}^{\it{K}} {\it{T} \times \alpha \times {\it{P}}_{\rm{0}} \times \left| {{\varvec{h}}_{k}^{\rm {H}} {{\varvec{\uptheta}}}{\varvec{g}}{ \rm{+} }h_{d,k}^{*} } \right|^{\rm 2} }$$

10 $${\text{s.t.}}\begin{array}{*{20}c} {} & {0 \le } \\ \end{array} \xi_{n} \le 2\pi ,\forall n \in {\varvec{N}}$$

It is worth noting that the hidden structure of Eq. (9) is fundamentally different from Eq. (5), i.e., **θ** can be configured only once, instead of flexibly adjusting for each individual node, as **θ** is shared by all CRSNs nodes. In addition, some nodes cannot harvest the maximum amount of energy at the optimal solution. As such, (P2) cannot be decomposed into *K* independent sub-problems to be solved optimally and independently, and the result in Eq. (7) is not applicable. This has non-trivial effects on the algorithm designed to solve the problem and calls for new algorithm design.

Problem (P2) is generally intractable due to its non-concave objective function, to be specific, its constraint is a convex function of *ξ*_*n*_, but the objective is non-concave, as such, (P2) is a non-convex problem. For a non-convex problem, there may exist multiple local optimal solutions in the feasible set, but the global optimal solution is unable to be quickly determined, i.e., exponential computational complexity is usually required to obtain the global optimal solution. As a result, non-convex problems are regarded as difficult and there is usually no standard method or algorithm to solve them. In this paper, SCA algorithm is applied to solve the non-convex problem (P2). SCA algorithm searches for a stationary point of the original problem by iteratively solving a convex problem which is similar to the original problem. To be specific, there are 4 basic steps:

1. A feasible point ***μ*** of the original function *f* is selected.
2. The approximate function f* is constructed for *f* based on ***μ*** to guarantee that f* and *f* share the same gradient at this point and f* has strong concavity.
3. By substituting the original objective function by f* and keeping the constraint unchanged, a next feasible point can be obtained by solving the newly formulated problem.
4. The above process is repeated until the convergence conditions are satisfied.

In order to exhibit the operation process of SCA algorithm clearly, the pseudo code is shown below:


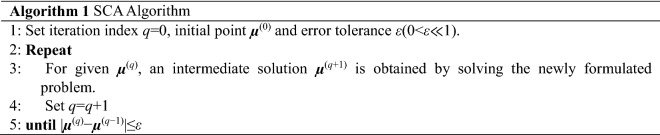


For the convenience of constructing the approximate function, the objective function in Eq. (9) should be simplified. As all CRSNs nodes share the same set of parameters *T*, *α* and *P*_0_, maximizing *E′*_*total*_ is completely equivalent to maximizing $\sum\limits_{k = 1}^{K} {\left| {{\varvec{h}}_{k}^{\rm {H}} {{\varvec{\uptheta}}}{\varvec{g}}{ + }h_{d,k}^{*} } \right|^{2} }$. In this case, the original optimization problem can be simplified into:

11 $$\rm {(P3}):\quad \mathop {\max }\limits_{{{\varvec{\uptheta}}}} \quad \sum\limits_{{\it{k}} = 1}^{\it{K}} {\left| {{\varvec{{h}}}_{\it{k}}^{\rm {H}} {{{\uptheta}}}{\varvec{{g}}}{ + }{\it{h}}_{{\it{d}},{\it{k}}}^{*} } \right|^{2} }$$

12 $${\text{s.t.}} \quad 0 \le \xi_{n} \le 2\pi ,\forall n \in {\varvec{N}}$$

Set $h_{d,k}^{*} = y_{k} ,\;{\varvec{h}}_{k}^{\rm {H}} {{\varvec{\uptheta}}}{\varvec{g}} = {\varvec{\mu}}^{\rm {H}} {\varvec{x}}_{k} ,$ here, ${\varvec{\mu}} = [\mu_{1} ,...,\mu_{n} ,...,\mu_{N} ]^{{\text{H}}} ,\;\mu_{n} = e^{{j}{\xi_{n} }} ,\;{\varvec{x}}_{k} = {\text{diag(}}{\varvec{h}}_{k}^{{\text{H}}} {)}{\varvec{g}}\;{.}$ The constraint in Eq. (12) can be equally transformed into unit module constraint |*μ*_*n*_|= 1. Correspondingly, the above optimization problem can be rewritten as:

13 $$\hbox{(P4}):\quad \mathop {\max }\limits_{{\varvec{\mu}}} \quad \sum\limits_{{\it{k}} = 1}^{\it{K}} {\left| {{\varvec{\mu}}^{\rm {H}} {{{\varvec{x}}}}_{\it{k}} + {{y_{k}}} } \right|^{2} }$$

14 $${\text{s.t.}} \quad \left| {\mu_{n} } \right| = 1,\forall n \in {\varvec{N}}$$

The objective function in Eq. (13) can be expanded as:

15 $$\sum\limits_{k = 1}^{K} {({\varvec{\mu}}^{{\text{H}}} {\varvec{x}}_{k} {\varvec{x}}_{k}^{{\text{H}}} {\varvec{\mu}} + {\varvec{\mu}}^{{\text{H}}} {\varvec{x}}_{k} y_{k} + {\varvec{x}}_{k}^{{\text{H}}} {\varvec{\mu}}y_{k} + \left| {y_{k} } \right|^{2} )}$$

Set ${\varvec{W}} = \sum\limits_{k = 1}^{K} {{\varvec{x}}_{k} {\varvec{x}}_{k}^{\rm {H}} }$, Eq. (15) can be transformed into:

16 $${\user2{\mu}}^{{\text{H}}} {\varvec{W}}\user2{\mu + }\sum\limits_{k = 1}^{K} {({\varvec{\mu}}^{{\text{H}}} {\varvec{x}}_{k} y_{k} + {\varvec{x}}_{k}^{{\text{H}}} {\varvec{\mu}}y_{k} + \left| {y_{k} } \right|^{2} )}$$

When SCA is applied, a feasible point $\widetilde{{{{\upmu}}}}$ of the original problem (P4) is randomly generated, and the first order Taylor expansion of (16) at $\widetilde{{{\varvec{\upmu}}}}$ is leveraged to construct its approximate function. According to the first order condition of convex function, its first order Taylor expansion at a given point is globally lower bounded. Therefore, the original objective function is globally lower bounded by its concave approximate function at $\widetilde{{{{\upmu}}}}$. In this case, the newly formulated problem is:

17 $$\rm {(P5}):\begin{array}{*{20}c} {\quad \mathop {\max }\limits_{{\varvec{\mu}}} } & {2{\text{Re}} \{ {\varvec{\mu}}^{\rm {H}} ({\varvec{W}}\widetilde{{\varvec{\mu}}} + \sum\limits_{k = 1}^{K} {{\varvec{x}}_{k} } y_{k}^{\rm {H}} )\} - \widetilde{{\varvec{\mu}}}^{\rm {H}} {\varvec{W}}\widetilde{{\varvec{\mu}}} + \sum\limits_{k = 1}^{K} {\left| {y_{k} } \right|^{2} } } \\ \end{array}$$

18 $${\text{s.t.}}\quad \left| {\mu_{n} } \right| = 1,\forall n \in {\varvec{N}}$$

From Eq. (17), it can be observed that the optimal μ^*^ is related to item ${\varvec{W}}\widetilde{{\varvec{\mu}}} + \sum\limits_{k = 1}^{K} {{\varvec{x}}_{k} } y_{k}^{\rm {H}}$, and the *n*th element of the next feasible solution is:

19 $$\mu_{n}^{*} { = }\left\{ \begin{gathered} 1\quad if\;\;{(}{\varvec{W}}\widetilde{{\varvec{\mu}}} + \sum\limits_{k = 1}^{K} {{\varvec{x}}_{k} y_{k}^{\rm {H}} } )_{n} = 0 \hfill \\ {(}{\varvec{W}}\widetilde{{\varvec{\mu}}} + \sum\limits_{k = 1}^{K} {{\varvec{x}}_{k} y_{k}^{\rm {H}} } )_{n} /\left| {{\varvec{W}}\widetilde{{\varvec{\mu}}} + \sum\limits_{k = 1}^{K} {{\varvec{x}}_{k} y_{k}^{\rm {H}} } } \right|_{n} \quad otherwise \hfill \\ \end{gathered} \right.$$

By comparing the obtained solution μ^*^ with $\widetilde{{{{\upmu}}}}$, if their difference is equal to or smaller than the predetermined error tolerance *ε* (0 < *ε* ≪ 1), μ^*^ is the final optimal solution of the original problem, otherwise a next feasible solution is iteratively generated from Eq. (19) by setting $\widetilde{{{{\varvec{\upmu}}}}} \rm{=} {{\varvec{\upmu}}}^{*}$.

The shared reflection coefficient matrix-based EH scheme obtains the optimal IRS reflection coefficient matrix by solving the corresponding optimization problem, and CRSNs nodes all harvest energy during time duration *T*. However, as the reflection coefficient of each IRS element should be determined, the complexity and time required to solve the optimization problem will increase as IRS elements or CRSNs nodes increase.

### Subsurface partition-based EH scheme for IRS-aided EH-CRSNs

**Figure 3:**
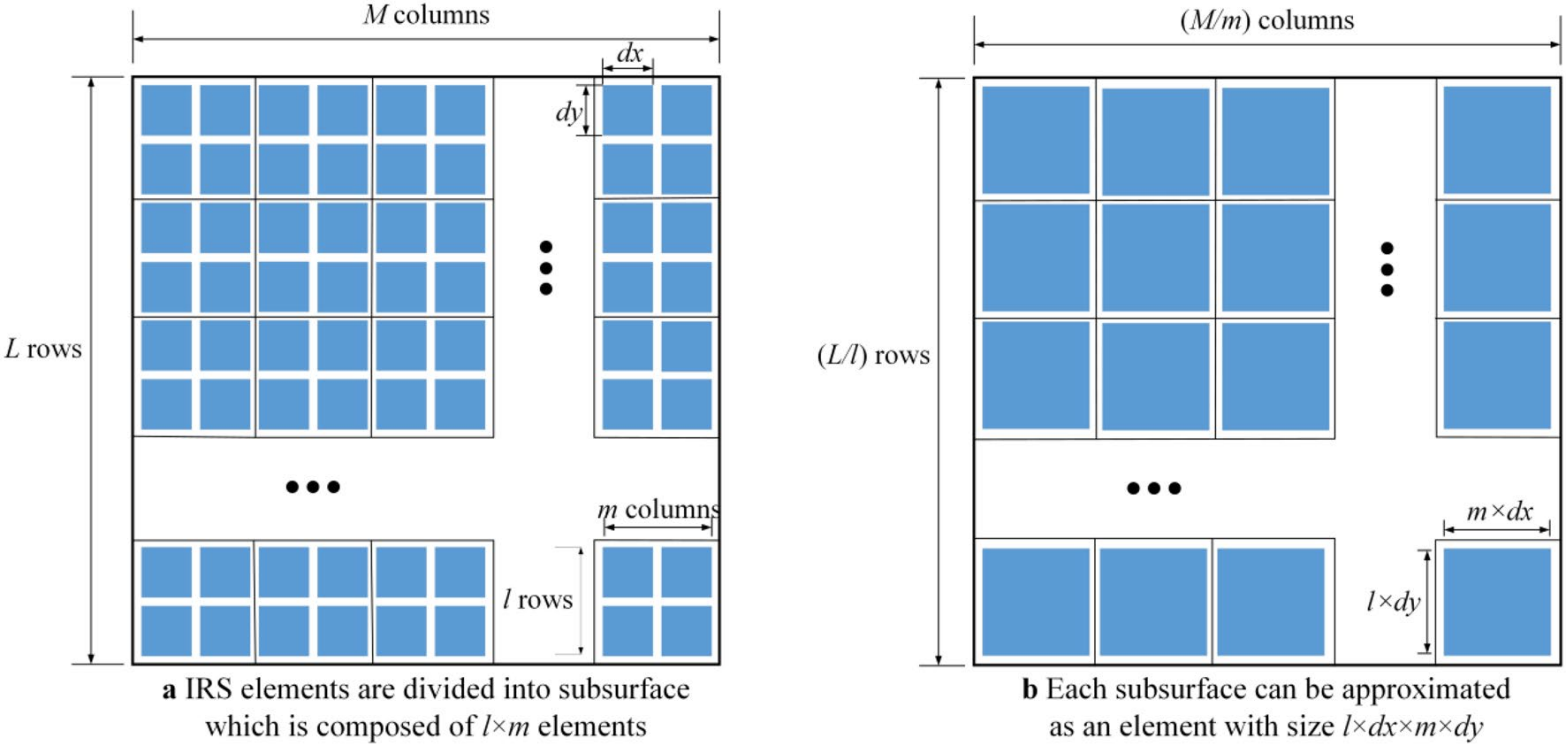
Subsurface partition of IRS.

To achieve a compromise between the total amount of harvested energy and complexity of solving the optimization problem, the subsurface partition-based EH scheme is proposed to divide all IRS elements into subsurface. All elements in the same subsurface constitute a new IRS element with larger size. As shown in Fig. 3, IRS is divided into *B* subsurface (denoted by set ***B***) which is composed of *l* rows and *m* columns, i.e., *B* = (*L*/*l*) × (*M*/*m*). All IRS elements on subsurface *b* (∀*b* ∈ ***B***) are assigned the same reflection coefficient, and the coordinate of its center is (*x*_*IRS_b*_,*y*_*IRS_b*_,*R*), where *b* = (*i − *1) × *M/m* + *j*, *i*(∈ [1,*L*/*l*]) and *j*(∈ [1,*M*/*m*]) represent the row and the column where subsurface *b* is located.

Based on the coordinate of IRS center and the size of each subsurface and element, the coordinate of the first subsurface center in the upper left corner can be determined, and then coordinates of other subsurface centers can also be figured out. The center of the first subsurface is located at (*x*_*IRS*_1_,*y*_*IRS*_1_,*R*) = (*x*_0_ − (*M* − *m*)*dx*/2,*y*_0_ + (*L* − *l*)*dy*/2,*R*), and that of subsurface *b* is (*x*_*IRS_b*_,*y*_*IRS_b*_,*R*) = (*x*_*IRS_*1_ + *m* × 

(*j* − 1)*dx*,*y*_*IRS_*1_ − *l* × (*i* − 1)*dy*,*R*).

According to the above coordinates, the Euclidean distance between the sink and node *k*, between the sink and the center of subsurface *b* and between the center of subsurface *b* and node *k* can be calculated as below:

20 $$d_{0}^{k} = \sqrt {(x_{k} - x_{0} )^{2} + (y_{k} - y_{0} )^{2} }$$

21 $$d_{1}^{b} = \sqrt {\left( {x_{{IRS\_1}} {\text{ + }}m \times {\text{(}}j - {\text{1)}}dx - x_{0} } \right)^{2} + \left( {y_{{IRS\_1}} - l \times {\text{(}}i - 1{\text{)}}dy - y_{0} } \right)^{2} + R^{2} }$$

22 $$d_{2}^{{b,k}} = \sqrt {\left( {x_{{IRS\_1}} {\text{ + }}m \times {\text{(}}j - {\text{1)}}dx - x_{k} } \right)^{2} + \left( {y_{{IRS\_1}} - l \times {\text{(}}i - 1{\text{)}}dy - y_{k} } \right)^{2} + R^{2} }$$

The equivalent channels between the sink and subsurface *b* and between subsurface *b* and node *k* are represented by *r*_*s*_*b*_ and *s*_*b*_*k*_, respectively. In this case, the equivalent composite channels between the sink and IRS and between IRS and node *k* can be denoted by ${\varvec{r}} \in {\mathbb{C}}^{B \times 1}$ and ${\varvec{s}}_{k}^{\rm {H}} \in {\mathbb{C}}^{1 \times B}$, respectively, and ***r*** = [*r*_*s*_1_,…,*r*_*s*_*b*_…,*r*_*s*_*B*_]^T^, ${\varvec{s}}_{k}^{\rm {H}} = [s_{1\_k}^{\rm {H}} ,...,s_{b\_k}^{\rm {H}} ...,s_{B\_k}^{\rm {H}} ]$. The IRS reflection coefficient matrix is denoted by **O** = diag(*O*_1_,…,*O*_*b*_,…,*O*_*B*_), where $O_{b} = \beta_{b} e^{{j\Omega }{_{b} }}$, *β*_*b*_ and *Ω*_*b*_ are the reflection amplitude and phase shift of subsurface *b*, respectively. For the subsurface partition-based EH scheme, the total energy harvested by all CRSNs nodes during *T* is:

23 $$E_{total}^{\prime \prime } = \sum\limits_{k = 1}^{K} {T \times \alpha \times P_{0} \times \left| {l \times m \times ({\varvec{s}}_{k}^{\rm {H}} {\mathbf{O}}{\varvec{r}}){ + }h_{d,k}^{*} } \right|^{2} }$$

The optimization problem which aims at maximizing the total amount of harvested energy can be formulated as:

24 $$\rm {(P6}):\quad \mathop {\max }\limits_{{\mathbf{O}}} \quad \sum\limits_{{\it{k}} = 1}^{\it{K}} {{\it{T}} \times \alpha \times {\it{P}}_{0} \times \left| {{\it{l}} \times {\it{m}} \times ({\varvec{{s}}}_{\it{k}}^{\rm {H}} {\mathbf{O}}{\varvec{{r}}}){ + }{\it{h}}_{{\it{d}},{\it{k}}}^{*} } \right|^{2} }$$

25 $${\text{s.t.}} \quad 0 \le \it\Omega_{b} \le {\rm2}\pi ,\forall b \in {\varvec{B}}$$

As all CRSNs nodes share the same set of parameters *T*, *α* and *P*_0_, maximizing *Etotal*′′ is completely equivalent to maximizing $\sum\limits_{k = 1}^{K} {\left| {l \times m \times ({\varvec{{s}}}_{k}^{\rm {H}} {\mathbf{O}}{\varvec{{r}}}){ + }h_{d,k}^{*} } \right|^{2} }$. In this case, (P6) can be simplified into:

26 $$\rm {(P7}):\quad \mathop {\max }\limits_{{\mathbf{O}}} \quad \sum\limits_{{\it{k}} = 1}^{\it{K}} {\left| {{\it{l}} \times {\it{m}} \times ({\varvec{{s}}}_{\it{k}}^{\rm {H}} {\mathbf{O}}{\varvec{{r}}}){ + }{\it{h}}_{{\it{d}},{\it{k}}}^{*} } \right|^{2} }$$

27 $${\text{s.t.}} \quad 0 \le {\it\Omega}_{b} \le 2\pi ,\forall b \in {\varvec{B}}$$

Set $h_{d,k}^{*} = y_{k}$ and $\;l \times m \times ({\varvec{s}}_{k}^{{\text{H}}} {\mathbf{O}}{\varvec{r}}) = {\varvec{\omega}}^{{\text{H}}} {\varvec{{z}}}_{k}$, here, ${{\varvec{\omega}}} = [\omega_{1} ,...,\omega_{b} ,...,\omega_{B} ]^{{\text{H}}} ,\;\omega_{b} = e^{{j\it{\Omega}_{b} }}$, ${\varvec{{z}}}_{k} = l \times m \times {\text{diag(}}{\varvec{s}}_{k}^{{\text{H}}} {)}{\varvec{{r}}}{,}\;\forall k$. The constraint in Eq. (27) can be equally transformed into unit module constraint |*ωb*| = 1. Correspondingly, the above optimization problem can be rewritten as:

28 $$\rm {(P8}):\quad \mathop {\max }\limits_{{\varvec{\omega}}} \quad \sum\limits_{{\it{k}} = 1}^{\it{K}} {\left| {{\varvec{\omega}}^{{\text{H}}} {\varvec{{z}}}_{\it{k}} + {\it{y}}_{\it{k}} } \right|^{2} }$$

29 $${\text{s.t.}} \quad \left| {\omega_{b} } \right| = 1,\forall b \in {\mathbf{B}}$$

The objective function in Eq. (28) can be expanded as:

30 $$\sum\nolimits_{{\it{k}} = 1}^{\it{K}} {\rm {(}{\varvec{\omega}}^{{\text{H}}} {\varvec{{z}}}_{\it{k}} {\varvec{{z}}}_{\it{k}}^{{\text{H}}} {\varvec{\omega}} + {\varvec{\omega}}^{{\text{H}}} {\varvec{{z}}}_{\it{k}} {\it{y}}_{\it{k}} + {\varvec{{z}}}_{\it{k}}^{{\text{H}}} {\varvec{\omega}}{\it{y}}_{\it{k}} + \left| {{\it{y}}_{\it{k}} } \right|^{2} } )$$

Set ${\varvec{U}} = \sum\limits_{k = 1}^{K} {{\varvec{{z}}}_{k} {\varvec{{z}}}_{k}^{\rm {H}} }$, Eq. (30) can be transformed into:

31 $${\varvec{\omega}}^{{\text{H}}} {\varvec{U}}{\varvec{\omega}}{ + }\sum\nolimits_{{\it{k}} = 1}^{K} {\rm {(}{\varvec{\omega}}^{{\text{H}}} {\varvec{{z}}}_{\it{k}} {\it{y}}_{\it{k}} + {\varvec{{z}}}_{\it{k}}^{{\text{H}}} {\varvec{\omega}}{\it{y}}_{\it{k}} + \left| {{\it{y}}_{\it{k}} } \right|^{2} } )$$

When SCA is applied, a feasible point $\widetilde{{\varvec{\omega}}}$ of the original problem (P8) is randomly generated, and the first order Taylor expansion of (31) at $\widetilde{{\varvec{\omega}}}$ is leveraged to construct its approximate function. According to the first order condition of convex function, its first order Taylor expansion at a given point is globally lower bounded. Therefore, the original objective function is globally lower bounded by its concave approximate function at $\widetilde{{\varvec{\omega}}}$. In this case, the newly formulated problem is:

32 $$\rm {(P9}):\quad \mathop {\max }\limits_{{\varvec{\omega}}} \quad 2{\text{Re}} \{ {\varvec{\omega}}^{{\text{H}}} ({\varvec{{U}}}\widetilde{{\varvec{\omega}}} + \sum\limits_{{\it{k}} = 1}^{\it{K}} {{\varvec{{z}}}_{\it{k}} } {\it{y}}_{\it{k}}^{H} )\} - \widetilde{{\varvec{\omega}}}^{{\text{H}}} {\varvec{{U}}}\widetilde{{\varvec{\omega}}} + \sum\nolimits_{{\it{k}} = 1}^{\it{K}} {\left| {{\it{y}}_{\it{k}} } \right|^{2} }$$

33 $${\text{s.t.}} \quad \left| {\omega_{b} } \right| = 1,\forall b \in {\mathbf{B}}$$

From Eq. (32), it can be observed that the optimal ω* is related to item ${\varvec{U}}\widetilde{{\varvec{\omega}}} + \sum\limits_{k = 1}^{K} {{\varvec{z}}_{k} } y_{k}^{{\text{H}}}$, and the *b*th element of the next feasible solution is:

34 $$\omega_{b}^{*} { = }\left\{ \begin{gathered} 1\quad if\;\;{(}{\varvec{U}}\widetilde{{\varvec{\omega}}} + \sum\limits_{k = 1}^{K} {{\varvec{{z}}}_{k} } y_{k}^{{\text{H}}} )_{b} = 0 \hfill \\ {(}{\varvec{U}}\widetilde{{\varvec{\omega}}} + \sum\limits_{k = 1}^{K} {{\varvec{{z}}}_{k} } y_{k}^{{\text{H}}} )_{b} /\left| {{\varvec{U}}\widetilde{{\varvec{\omega}}} + \sum\limits_{k = 1}^{K} {{\varvec{{z}}}_{k} } y_{k}^{{\text{H}}} } \right|_{b} \quad otherwise \hfill \\ \end{gathered} \right.$$

By comparing the obtained solution ω* with $\widetilde{{\varvec{\omega}}}$_,_ if their difference is equal to or smaller than the predetermined error tolerance *ε*, ω* is the final optimal solution of the original problem, otherwise a next feasible solution is iteratively generated from Eq. (34) by setting $\widetilde{{\varvec{\omega}}} =$ ω*. The optimal IRS reflection coefficient obtained for each subsurface is shared by all elements on it. The actual total amount of energy harvested by all CRSNs nodes can be derived by substituting the obtained reflection coefficients into Eq. (8).

## Results and discussion

To test the performance of various EH schemes proposed for IRS-aided EH-CRSNs in this paper, Matlab is leveraged to compare the amount of harvested energy. The sink is located at (0,0,0), and its transmission power is set as 40 W^34,35^.The conversion efficiency of EH circuits *α* is set as 0.8 which is a commonly used parameter configuration in linear EH models^36–38^. IRS is usually configured with thousands of elements with sub-wavelength size to achieve its array gain. Here, the width of each IRS element is set as less than one half of the wavelength of transmission signal. The coordinate of IRS center is (0,0,5), and its other parameter settings are the same as in^39^. The detailed simulation parameters settings are listed in Table 2.

**Table 2:** Simulation parameter settings.

Parameters	Values
Network radius *A*	100 m
Total number of CRSNs nodes *K*	100
Transmission Power of the sink *P*_0_	40 W
Number of rows on IRS *L*	34
Number of columns on IRS *M*	50
Size of each IRS element *dx* × *dy*	0.01 m × 0.01 m
Wavelength of transmission signal *λ*	0.0286 m
Time duration of EH *T*	1 s
Conversion efficiency of EH circuits *α*	0.8

**Figure 4:**
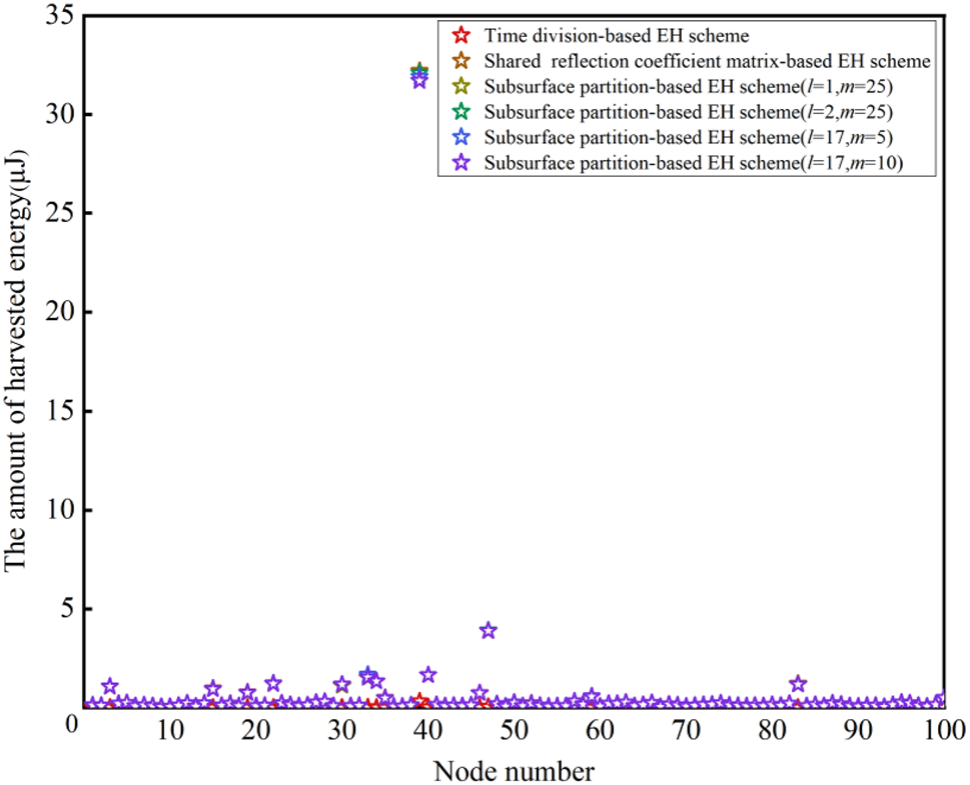
Comparison results of the amount of energy harvested by each CRSNs node.

In subsurface partitioned-based EH schemes, IRS is divided into multiple regular subsurface, and each of them is composed of *l* rows and *m* columns. This requires that *L* should be divisible by *l* and *M* should be divisible by *m*. In this case, *l* can be set to 1, 2, 17 and 34 while *m* can be set as 1, 2, 5, 10, 25 and 50. Different values of *l* and *m* can be combined to produce 24 subsurface partition-based EH schemes, and we choose 4 representative schemes among them to illustrate the impact of the manner of subsurface partition: (1) *l* = 1, *m* = 25 means that IRS is divided into (34/1) × (50/25) = 68 subsurface. (2) *l* = 2, *m* = 25 represents that IRS is partitioned into (34/2) × (50/25) = 34 subsurface. (3) *l* = 17, *m* = 5 means that IRS is divided into (34/17) × (50/5) = 20 subsurface. (4) *l* = 17, *m* = 10 represents that IRS is partitioned into (34/17) × (50/10) = 10 subsurface. The amount of energy harvested by each CRSNs node is recorded, and the results are shown in Fig. 4.

**Figure 5:**
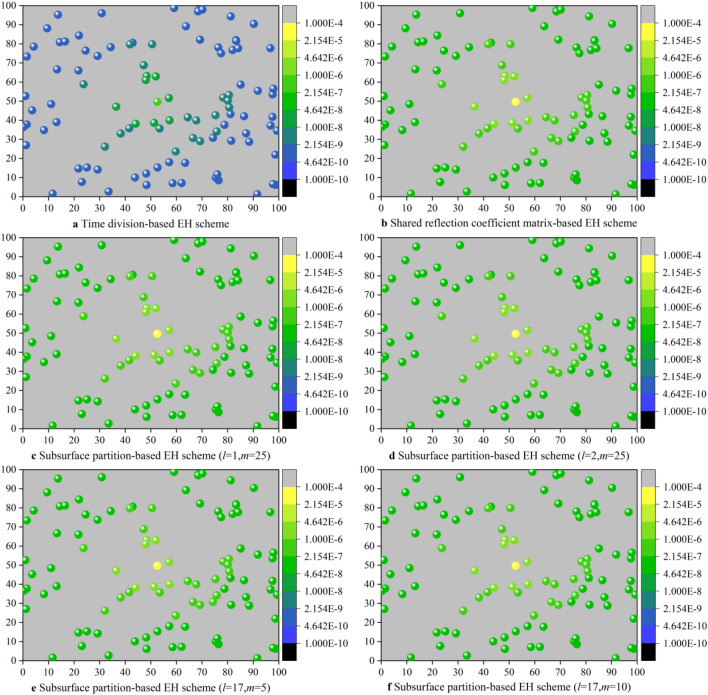
Heat maps for exhibiting the amount of energy harvested by each CRSNs node in different EH schemes.

Compared with the time division-based EH scheme, the shared reflection coefficient matrix-based EH scheme and the subsurface partition-based EH schemes enable each CRSNs node to harvest much more energy. The reasons are analyzed as follows: (1) The time division-based EH scheme divides the EH duration evenly into 100 time slots, and each CRSNs node is only allowed to harvest energy within its dedicated time slot. As the amount of harvested energy is positively proportional to the EH duration, the energy harvested by each CRSNs node is still very limited even though IRS is optimally configured to serve the node. (2) In the shared reflection coefficient matrix-based EH scheme and the subsurface partition-based EH schemes, although the optimal IRS reflection coefficient matrix cannot guarantee that each CRSNs node can harvest the maximum amount of energy, all CRSNs nodes can make full use of the total EH duration *T* to harvest RF energy from the sink. It should be noted that node 39 which is the closest to the sink can harvest more energy than others in all EH schemes. There are 2 reasons: Firstly, the Euclidean distance between node 39 and the sink is the smallest, which means smaller path loss, correspondingly, more energy can be harvested; Secondly, the optimization problems are formulated with the objective of maximizing the total amount of harvested energy. As the energy balance among nodes is not taken into consideration, the optimal IRS reflection coefficient matrix is more in favor of node 39. To validate the above analysis, the amount of energy harvested by CRSNs nodes at different locations is exhibited in the heat map shown in Fig. 5. In addition, as can be observed from Fig. 4, the harvested energy of the 4 subsurface partition-based EH schemes exhibits basically the same trend. However, the amount of energy harvested by each node decreases as the number of subsurface reduces. As all elements in the same subsurface share identical reflection coefficient, compared with the EH scheme which optimizes reflection coefficient for each element, distance between all elements on the subsurface and the sink is completely denoted by the distance from the subsurface center to the sink. In this case, reflection coefficients may be inaccurate so that the gains brought by IRS get smaller.

**Figure 6:**
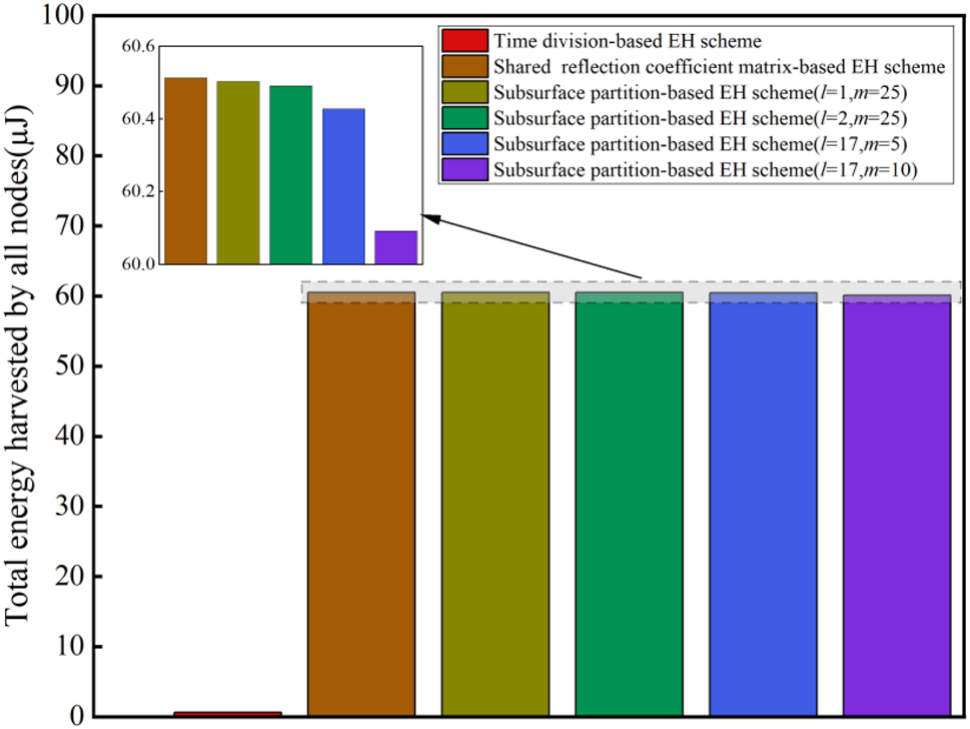
Comparison results of the total amount of harvested energy.

To further quantitatively evaluate various EH schemes, the total amount of energy harvested by all CRSNs nodes is shown in Fig. 6. It can be observed that there is a big gap among the EH schemes proposed in this paper. By optimizing IRS reflection coefficient matrix, the shared reflection coefficient matrix-based EH scheme and the subsurface partition-based EH schemes enable each CRSNs node to fully leverage the EH duration and enjoy the benefits brought by IRS. In addition, the total amount of energy harvested by the 4 subsurface partition-based EH schemes is 0.015%, 0.096%, 0.140% and 0.696% lower than the shared reflection coefficient matrix-based EH scheme, respectively, i.e., the performance loss caused by subsurface partition is relatively small. On the other hand, subsurface partition can dramatically reduce the complexity of deriving the optimal IRS reflection coefficient matrix. Therefore, the subsurface partition-based EH schemes are compromise solutions to achieve a good balance between EH performance and computational complexity.

## Conclusions

Focusing on the low energy utilization ratio problem in legacy EH-CRSNs, IRS is introduced to form IRS-aided EH-CRSNs, and EH schemes are designed to increase the amount of harvested energy. The non-convex optimization problem with the objective of maximizing the total amount of energy harvested by all CRSNs nodes is formulated. SCA algorithm is leveraged to obtain the optimal IRS reflection coefficient matrix configuration, and the computational complexity can be further reduced by subsurface partition. Simulation results show that there is a big difference in the amount of energy harvested by nodes, and the reasons are analyzed as follows: different Euclidean distance to the sink results in distinguished signal propagation loss, and the IRS reflection coefficient matrix configuration will cater more to nodes close to the sink. In addition, the subsurface partition-based EH schemes substitute the distance between various elements and CRSNs nodes/the sink with that between the subsurface center and CRSNs nodes/the sink, which will result in error. Correspondingly, compared with the shared reflection coefficient matrix-based EH scheme, the subsurface partition-based EH schemes will introduce less than 1% performance loss, and the performance loss will decrease as the number of subsurface increases. However, the EH schemes proposed in this paper leave EH fairness out of consideration, which may result in unbalanced energy distribution among nodes and shorten the network lifetime. In addition, simple linear EH model is leveraged to quantify the harvested energy and simplify the formulated optimization problems. In this case, the upper limit of EH performance achieved by IRS-aided EH-CRSNs is exhibited. In fact, non-linear components in practical EH circuits will lead to the non-linear characteristic of end-to-end energy conversion, and non-linear EH models are more accurate. In our future work, we will propose new EH schemes to further consider about non-linear EH models and balancing the energy harvested by CRSNs nodes.

## Data Availability

The datasets generated during and/or analyzed during the current study are available from the corresponding author on reasonable request.
